# High-density genotyping reveals candidate genomic regions for chicken body size in breeds of Asian origin

**DOI:** 10.1016/j.psj.2022.102303

**Published:** 2022-10-29

**Authors:** Shijie Lyu, Danny Arends, Mostafa K. Nassar, Annett Weigend, Steffen Weigend, Eryao Wang, Gudrun A. Brockmann

**Affiliations:** ⁎ Albrecht Daniel Thaer-Institute of Agricultural and Horticultural Sciences, Humboldt-Universitt zu Berlin, Berlin 10115, Germany; † Institute of Animal Science and Veterinary Medicine, Henan Academy of Agricultural Sciences, Zhengzhou 450002, China; ‡ Department of Applied Sciences, Northumbria University, Newcastle upon Tyne, UK; § Animal Production Department, Faculty of Agriculture, Cairo University, Giza 12613, Egypt; # Institute of Farm Animal Genetics, Friedrich-Loeffler-Institut, Neustadt-Mariensee 31535, Germany

**Keywords:** Asian Game type chicken, Asian Bantam type chicken, growth, GWAS, PCA

## Abstract

Body size is one of the main selection indices in chicken breeding. Although often investigated, knowledge of the underlying genetic mechanisms is incomplete. The aim of the current study was to identify genomic regions associated with body size differences between Asian Game and Asian Bantam type chickens. In this study, 94 and 107 chickens from 4 Asian Game and 5 Asian Bantam type breeds, respectively, were genotyped using the chicken 580K single nucleotide polymorphism (**SNP**) array. A genome-wide association study (**GWAS**) and principal component analyses (**PCA**) were performed to identify genomic regions associated with body size related-traits such as wing length, shank length, shank thickness, keel length, and body weight. Hierarchical clustering of genotype data showed a clear genetic difference between the investigated Asian Game and Asian Bantam chicken types. GWAS identified 16 genomic regions associated with wing length (2, FDR ≤ 0.018), shank thickness (6, FDR ≤ 0.008), keel length (5, FDR ≤ 0.023), and body weight (3, FDR ≤ 0.041). PCA showed that the first principal component (PC1) separated the 2 chicken types and significantly correlated with the measured body size related-traits (*P* ≤ 2.24e-40). SNPs contributing significantly to PC1 were subjected to a more detailed investigation. This analysis identified 11 regions potentially associated with differences in body size related-traits. A region on chromosome 4 (**GGA4**) (17.3–21.3 Mb) was detected in both analyses GWAS and PCA. This region harbors 60 genes. Among them are myotubularin 1 (***MTM1***) and secreted frizzled-related protein 2 (***SFPR2***) which can be considered as potential candidate genes for body size related-traits. Our results clearly show that the investigated Asian Game type chicken breeds are genetically different from the Asian Bantam breeds. A region on GGA4 between 17.3 and 21.3 Mb was identified which contributes to the phenotypic difference, though further validation of candidate genes is necessary.

## INTRODUCTION

The genetic mechanisms underlying variation in chicken body size is still insufficiently understood. Chicken body size reflects body development, especially bone and muscle growth (Gao et al., 2011; Geng et al., 2021). Growth is one of the main selection criteria in chicken breeding. Genetically, chicken body size is affected by many genes on the autosomes and the sex-chromosome, making it a truly complex trait. Hundreds of quantitative trait loci (**QTL**) have been mapped on autosomes for body size related-traits such as shank length, keel length, and body weight (Gao et al., 2011; Hu et al., 2012; Lyu et al., 2017; Johnsson et al., 2018; Mebratie et al., 2019; Dadousis et al., 2021). On the Z chromosome, for example, a mutation in the growth hormone receptor (***GHR***) gene results in sex-linked dwarfism (Agarwal et al., 1994). Many mapping studies used commercial chicken populations (Hu et al., 2012; Mebratie et al., 2019; Dadousis et al., 2021). In such populations, genetic loci are detected which account significantly to the variance of the examined population. However, natural variation contributing to body size related-traits cannot be detected completely using this approach. For a more comprehensive understanding of the genetic determination of growth, the identification of additional genetic variants that contribute to body size in other wild populations is desireable. Thus, the investigation of genetically more diverse populations could help to identify additional genetic variants with influence on body size.

Besides mapping studies, comparative genomic analyses within and among different chicken breeds have been successfully used to identify signatures of selection and candidate genes for body size (Rubin et al., 2010; Wang et al., 2016, 2017). Since selection results in an increase of the frequency of beneficial alleles within a breed up to fixation the history of selection leaves behind selection signatures in the genome around the gene(s) that contributed to the selection response (Walugembe et al., 2018). However, when comparing breeds, not only the trait of interest (e.g., body weight) may be responsible for these signatures of selection, other traits that characterize a breed (e.g., the comb shape) can also cause such signatures of selection.

With respect to traits, breeds with similar genetic origin, selection goal (e.g., high body weight), and selection pressure can be grouped together in what is called a chicken type (Weigend et al., 2014). Comparing different chicken types should allow us to more accurately detect signatures of selection which are associated with a trait of interest, while minimizing signatures of selection originating from other traits. For example, other traits (e.g., comb shape) than the primary trait of interest (e.g., body size) can differ between the breeds belonging to one chicken type, while the only trait that is consistently different between 2 chicken types is the trait of interest.

Game and Bantam are 2 chicken types with Asian origin which differ considerably in body size (Weigend et al., 2014). Since Asian Game type chickens were selected for cock fighting ability, they are all tall and muscular. In contrast, Asian Bantam type chickens were selected for small body size. The two extreme chicken types were used in the current study with the aim to identify genetic factors underling the difference in body size. Therefore, we performed a genome-wide association study (**GWAS**) followed by a principal component analysis (**PCA**) using body size related-traits and high-density single nucleotide polymorphism (**SNP**) data.

## MATERIALS AND METHODS

### Animals and Phenotypes

A total of 201 chickens from 9 breeds were used in this study. According to the European Poultry Standards (Bund Deutscher Rassegeflügelzüchter, 2006) 4 breeds are classified as Asian Game type chickens [Aseel red mottled (**ASrb**, n = 19), Malay black red (**MAxx**, n = 30), Orloff red spangled (**OFrbx**, n = 25), Indian Game dark (**IKxx**, n = 20)] and 5 breeds as Asian Bantam type chickens [Japanese Bantam black tailed buff (**CHgesch**, n = 22), Japanese Bantam black mottled (**CHschw**, n = 29), Ko Shamo black-red (**KSgw**, n = 20), Ohiki red duckwing (**OHgh**, n = 18), Ohiki silver duckwing (**OHsh**, n = 18)] (Table 1, Figure S1). In total, we had 94 Asian Game type and 107 Asian Bantam type chickens. For each breed, nearly equal numbers of males and females were available. Wing length, shank length, shank thickness, keel length, and body weight were measured for every individual according to the protocol used in the Institute of Farm Animal Genetics at Friedrich-Loeffler-Institute (Table S1). All phenotypic data used in this study were available from the “Synbreed Chicken Diversity Panel”, which was collected within the framework of the SYNBREED project and according to SYNBREED protocols (Weigend et al., 2014; Malomane et al., 2019).

**Table 1:** Number of chickens in each breed.

Type	Breed	Number of individuals	
		Males	Females
Asian Game type	Aseel red mottled (ASrb)	9	10
	Malay black red (MAxx)	15	15
	Orloff red spangled (OFrbx)	12	13
	IKxx (Indian Game dark)	10	10
Asian Bantam type	Japanese Bantam black tailed buff (Chgesch)	10	12
	Japanese Bantam black mottled (CHschw)	13	16
	Ko Shamo black-red (KSgw)	10	10
	Ohiki red duckwing (OHgh)	8	10
	Ohiki silver duckwing (OHsh)	10	8

### Genotypes

Blood collection and DNA extraction were performed according to the SYNBREED protocol (Weigend et al., 2014; Malomane et al., 2019). Single nucleotide polymorphism (**SNP**) genotyping was performed using the Affymetrix Axiom Genome-Wide Chicken Genotyping Arrays encompassing over 580K SNPs (Kranis et al., 2013). The position of each SNP was based on the *Gallus gallus* 5 reference genome (Galgal5, Ensembl 91). SNPs were excluded using the following criteria across all chickens: 1) SNPs with call rates < 95%; 2) SNPs monomorphic across all breeds; 3) SNPs with a minor allele frequency (**MAF**) < 0.05. After quality control, 373,514 SNPs remained for hierarchical cluster analysis.

For genome-wide analysis, the genotypes of a genotype class of a specific marker were set to missing data, if the genotype class contained less than 10 individuals. This was done to prevent spurious associations in the GWAS. Afterward, markers were rechecked using the same procedure as outlined above. After filtering, 352,863 SNPs remained for GWAS. Since PCA requires full genotype data, markers containing missing data were removed leading to 172,737 SNPs suitable for PCA.

### Hierarchical Clustering

Hierarchical clustering of genotypes was performed to measure genetic distances between the 9 chicken breeds and to identify outliers. Outliers are defined as individuals which clustered within the wrong breed. Hierarchical clustering was carried out by calculating distances between individuals using Euclidean distance based on their genotypes. Afterward, the “hclust” function in R (3.2.3) (R Core Team, 2007) was used to cluster individuals using complete-linkage. A dendrogram was generated to visualize the relationship between individuals, and identify outliers.

### Phenotype Analysis

The two-tailed Student's *t* test was used to compare mean values of each trait and sex between the 2 chicken types. The significance threshold after standard Bonferroni correction for multiple testing was set to a *P-*value of 0.005 (0.05/(N_Traits(5)_ * N_Sexes(2)_)).

### Genome-Wide Association Study

Before performing the GWAS each phenotype was corrected for sex differences within each breed using the following linear model:

Y=μ+Sex+e,

Then, we took the intercept and summed it with the residuals after model fitting to be the new corrected phenotype data (Y’). Association analysis between the corrected phenotype and SNP genotype data was performed using R (3.2.3) with the following linear model:

$$Y^{\prime }=\mu +Breed+SNP+e,$$

where Y’ is the phenotype data corrected for the breed specific sex effect, µ is the population mean, breed (9 levels) accounts for the breed of the animal, and SNP is the SNP under investigation (coded as AA, AB, and BB). The *P*-values were corrected for multiple testing by estimating the false discovery rate (**FDR**) using the Benjamini-Hochberg procedure (Benjamini et al., 1995; Noble, 2009). SNPs with an FDR < 0.05 were considered significant. Variance explained by a SNP was calculated by dividing the difference of the residual sum of squares of the full model (with SNP effect) and the reduced model (without SNP effect) by the residual sum of squares of the reduced model (Brockmann et al., 2000). Additionally, genomic inflation factors (λ) were calculated by dividing the median of the observed *P*-values by the expected median from a χ^2^ distribution with one degree of freedom (Yang et al., 2011).

When estimating the additive effect of a SNP, the major homozygous, heterozygous, and minor homozygous genotypes were numerically coded as 0, 1, and 2, respectively. The regression coefficient (*β*) of a SNP effect represents the additive effect of the minor allele of this SNP (Parker et al., 2011; Lyu et al., 2014). For significant SNP, pairwise linkage disequilibrium (**LD**) with neighboring SNPs was calculated as r^2^ values using the “genetics” R package (Warnes et al., 2013). Adjacent significant SNPs were merged into one genomic region if they were in strong LD (r^2^ > 0.5) (Pattaro et al., 2016). Furthermore, the overlap between the identified associated regions and previously mapped QTLs for body size related traits in chicken was determined using data from the animal QTL database (QTLdb, Release 34) (Hu et al., 2012).

### Principal Component Analysis

In another approach, the genetic relationship between Asian Game and Bantam chicken types was analyzed by PCA using the “prcomp” function in R (Ritter et al., 2007). PCA can be used to identify principal components (**PCs**) which capture the majority of the genetic variation observed among genotypes (Rahmatalla et al., 2017). SNPs contributing significantly to the first principal component (**PC1**) were further investigated to identify those SNPs that allowed the PC1 to separate the 9 chicken breeds. The method for calculating the contribution of each SNP to a certain PC has been described previously (Rahmatalla et al., 2017). In brief, to select the SNPs with significant contribution, variable correlations with PC1 were calculated by multiplying the loading factors with the principal component standard deviation. The quality of representation for variables on the factor map (cos^2^) was calculated as the squared variable correlation. Afterward, all the cos^2^ values for PC1 were summed up. Contribution of each SNP was expressed as the percentage of total variation.

Since the original method does not allow for estimating significance of the SNP contribution, the method was extended by taking the sign of the PC1 rotation for each marker and multiplying it with its variable contribution to produce a “PCA observe distance measure” (**pcaDObs**). Afterward, the pcaDObs measurements were converted to *P*-values to identify SNPs significantly contributing to PC1 using the R “pnorm*”* function with the following equation:

p−value=2*pnorm(|pcaDObs|,mean=meanofpcaDObs,sd=SDofpcaDObs,lower.tail=FALSE)

where pnorm is the distribution function for the normal distribution (converts an observed value from a normal distribution to the corresponding *P*-value). The mean and standard deviation (**SD**) of the observed distribution were given to allow conversion of the observed values. The “lower.tail = FALSE” parameter meant that probabilities were defined as: P[X > x]. This approach allowed the identification of those SNPs capturing a significant differentiation among breeds which are separated by a given PC. In our analysis, only PC1 was investigated since this PC separated Asian Game from Asian Bantam type chickens. The *P*-values were adjusted for multiple testing using Bonferroni correction (P_Bonf_). SNPs with P_Bonf_ < 0.05 were considered as significant contributors to PC1. Compared with the previous approach which selected SNPs with more than 10 times the expected contribution to a given PC (Rahmatalla et al., 2017), our approach provides a statistical way to select SNPs significantly contributing to a certain PC by setting a statistical significance threshold instead of an arbitrary threshold.

SNPs were merged into a region when SNPs are located within 2 Mb of each other. In addition, for each PCA region the overlap was checked with known QTLs available in QTLdb (Release 34) (Hu et al., 2012).

### Investigation of Positional Candidate Genes

Genes within the identified candidate region(s) were extracted and investigated for potential functional association with body size. Gene function and its associated gene ontology (**GO**) terms and biological pathways were investigated using the “Functional Annotation Table” tool in the DAVID Bioinformatics Resources 6.8 (Huang et al., 2009a,b). Genes having roles in growth, bone or muscle development were prioritized. Moreover, to check the expression pattern of the prioritized candidate gene(s), the public databases “Expression Atlas” (Papatheodorou et al., 2018) and “BioGPS” (Wu et al., 2009) were used.

## RESULTS

### Hierarchical Clustering

**Figure 1:**
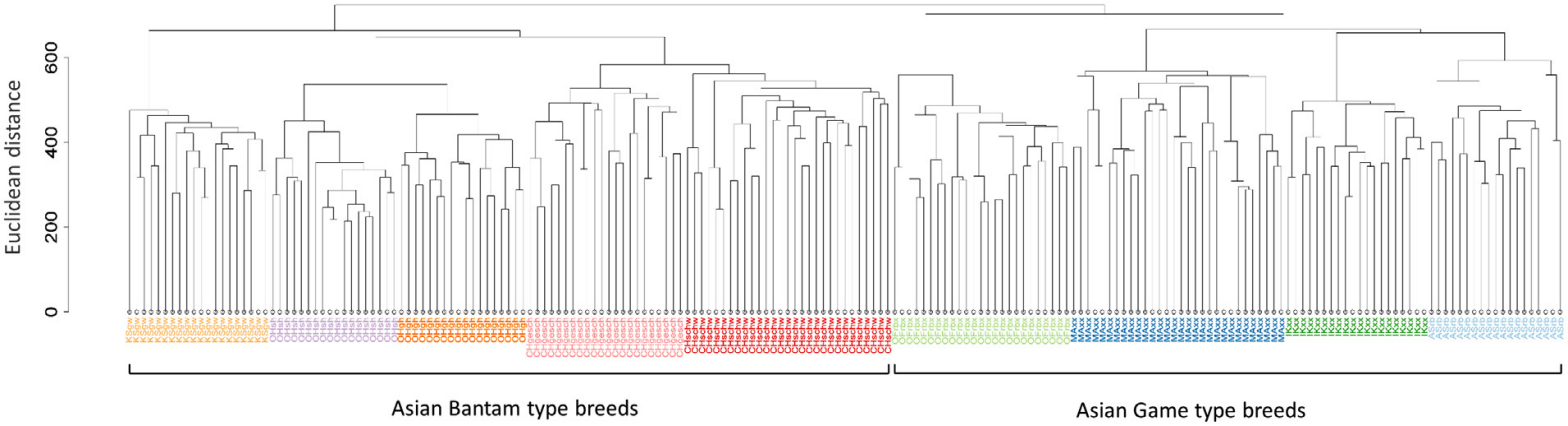
Hierarchical clustering of all chickens.

Hierarchical clustering of the genotype data showed 2 distinct clusters; one for Asian Game type and another for Asian Bantam type chickens (Figure 1), which indicated that the 2 chicken types have significant genetic differences. The breeds were well separated indicating that it is possible to assign the breed of an individual based on its genotype data.

### Phenotypic Characteristics

Asian Game type and Asian Bantam type chickens showed significant phenotypic differences on all measured traits (wing length, shank length, shank thickness, keel length, and body weight), in males as well as in females (*P* ≤ 1.23e-21, Figure S2). For all measured traits, Asian Game type chickens are significantly larger than Asian Bantam type chickens.

### Genome-Wide Association Study

**Figure 2:**
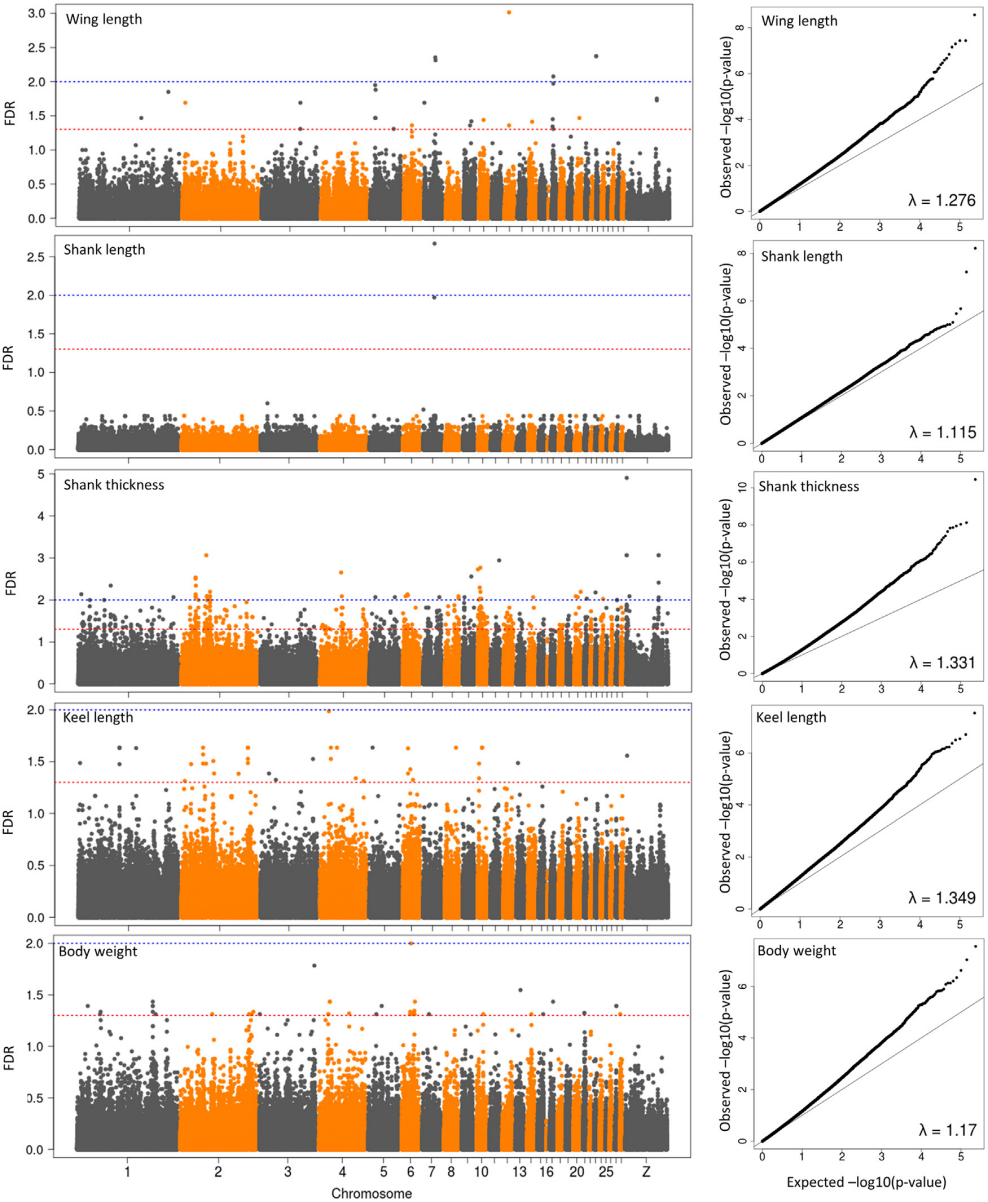
Manhattan plot and quantile-quantile plots of genome-wide association analyses for different traits. Y-axis represents the false discovery rate (FDR) of SNPs using Benjamini-Hochberg correction for multiple testing in GWAS. The blue and red dashed lines correspond to the 1% and 5% significance thresholds, respectively.

GWAS identified 622 SNPs significant for wing length (30), shank length (2), shank thickness (507), keel length (42), and body weight (41) (FDR < 0.05; 1.12 ≤ λ ≤ 1.35, Figure 2). Since most markers were detected for shank thickness, the markers with FDR < 0.01 were first analyzed for this trait, which led to 52 remaining markers. These highly significant markers for the measured traits were located in 16 regions on chicken chromosomes (**GGA**) 1, 2, 4, 6, 10, 23, 27, and Z (Table 2). We found 2 QTL for wing length on GGA23 and Z; 6 QTL for shank thickness on GGA2,10, and Z; 5 QTL for keel length on GGA1, 2, 4, 6, and 10; and 3 QTL for body weight on GGA1, 4, and 27. The region on GGA4 from 17,284,783 to 21,270,248 bp was found to be associated with both keel length and body weight. Within this region, the top SNP for keel length was SNP *rs314042510* (GGA4: 17,284,783, FDR = 0.010, MAF = 0.12) which showed an additive effect of −0.918 cm per C allele. In the same region, the top SNP for body weight was SNP *rs314732179* (GGA4: 21,243,556, FDR = 0.037, MAF = 0.29) which had an additive effect of 0.227 kg per A allele. Out of the 16 identified regions, the 3 identified regions on GGA6, 10, and 27 for keel length, shank thickness, and body weight, respectively, overlapped with QTLs that had been identified for similar traits in other populations (Table 2).

**Table 2:** Significant results of the genome-wide association study.

Trait	Top SNP^1^	Region^2^	Genotypes (number of individuals)	Minor allele	MAF^3^	Raw *P-*-value^4^	FDR^5^	*β* (SE)^6^	Var %^7^	Overlap with QTLs published in QTLdb^8^
Wing length (cm)	rs318160403	23: 97236 - 98116	GG (54), AG (79), AA (68)	G	0.47	3.63E-08	0.004	0.232 (0.039)	16.5	-
	rs316879242	Z: 57658911 - 57666420	GG (11), AA (180)	G	0.06	5.50E-07	0.018	0.316 (0.061)	15.7	-
Shank thickness (mm)	rs314712355	2: 27742242 - 27760815	CC (15), CT (15), TT (171)	C	0.11	1.00E-07	0.003	0.555 (0.134)	15.6	-
	rs314245637	2: 28143942 - 28240351	GG (78), AG (23), AA (100)	G	0.45	2.09E-07	0.005	−0.412 (0.118)	15.0	-
	rs316969703	2: 50086804 - 50116697	GG (23), AG (12), AA (166)	G	0.14	6.61E-07	0.008	−0.587 (0.120)	13.9	-
	rs15997785	2: 55910522 - 55928488	AA (50), AG (26), GG (123)	A	0.32	3.63E-07	0.006	−0.457 (0.095)	14.8	-
	rs315729689	10: 4534620 - 7754637	TT (25), CT (19), CC (157)	T	0.17	3.89E-08	0.002	−0.157 (0.167)	16.4	Body weight and Blood glucose level (95422 and 71191, broiler x Fayoumi)
	rs14773049	Z: 63407861 - 63418506	AA (85), AG (12), GG (104)	A	0.45	1.38E-08	0.001	−0.342 (0.110)	17.3	-
Keel length (cm)	rs315730958	1: 80330867 - 80373370	AA (47), AG (64), GG (90)	A	0.39	1.95E-07	0.023	−0.304 (0.101)	15.0	-
	rs14251327	2: 129987598 - 130043402	AA (18), AG (24), GG (159)	A	0.15	3.16E-07	0.023	0.222 (0.112)	14.6	-
	rs314042510	4: 17284783 - 21270248	CC (15), CT (18), TT (167)	C	0.12	2.95E-08	0.010	−0.918 (0.148)	16.8	-
	rs315991717	6: 10926588 - 10933626	AA (12), AC (27), CC (162)	A	0.13	1.15E-06	0.023	−0.679 (0.142)	13.4	Feed conversion ratio (139,402, Marshall breed, an indigenous broiler line from India.), Tibia bone mineral content and Tibia volume (135,879 and 135,882, meat-type chicken lines)
	rs315851423	10: 9230030 - 9231656	GG (11), AG (11), AA (177)	G	0.08	8.51E-07	0.023	−0.447 (0.218)	15.1	-
Body weight (kg)	rs318014118	1: 147653720 - 147680675	GG (17), AG (32), AA (152)	G	0.16	8.32E-07	0.037	0.143 (0.037)	13.7	-
	rs314732179	4: 18316381 - 21250309	AA (45), AG (25), GG (131)	A	0.29	7.41E-07	0.037	0.227 (0.049)	13.8	-
	rs312580626	27: 3375301 - 3375436	TT (21), CT (38), CC (141)	T	0.20	1.62E-06	0.041	0.201 (0.043)	13.2	Femur bone mineral content and Femur weight (130,479 and 130,480, Chinese indigenous breed × White Leghorn)

1 The SNP with the lowest *P*-value in the genome-wide association analysis for the relevant trait in a defined region.

2 The defined region associated with the relevant trait. The regions were defined by merging adjacent significant SNPs that were in strong LD (r^2^ > 0.5). Regions were shown as “chromosome: region start position – region end position”. The chromosome locations are based on Galgal5 (Ensembl 91).

3 MAF: minor allele frequency of the top SNP.

4 The *p*-value of the top SNPs without correction for multiple testing.

5 The *p*-value of the top SNPs were corrected for multiple testing by estimating the FDR using the Benjamini and Hochberg method.

6 *β* corresponds to the effect size of the minor allele of the top SNP on phenotype corrected for sex differences per breed.SE: standard error.

7 Percentage of variance explained by the top SNP.

8 Previously identified QTLs with records in the QTLdb (https://www.animalgenome.org/cgi-bin/QTLdb/index). IDs of the QTLs were shown in brackets.

### Principal Component Analysis

**Figure 3:**
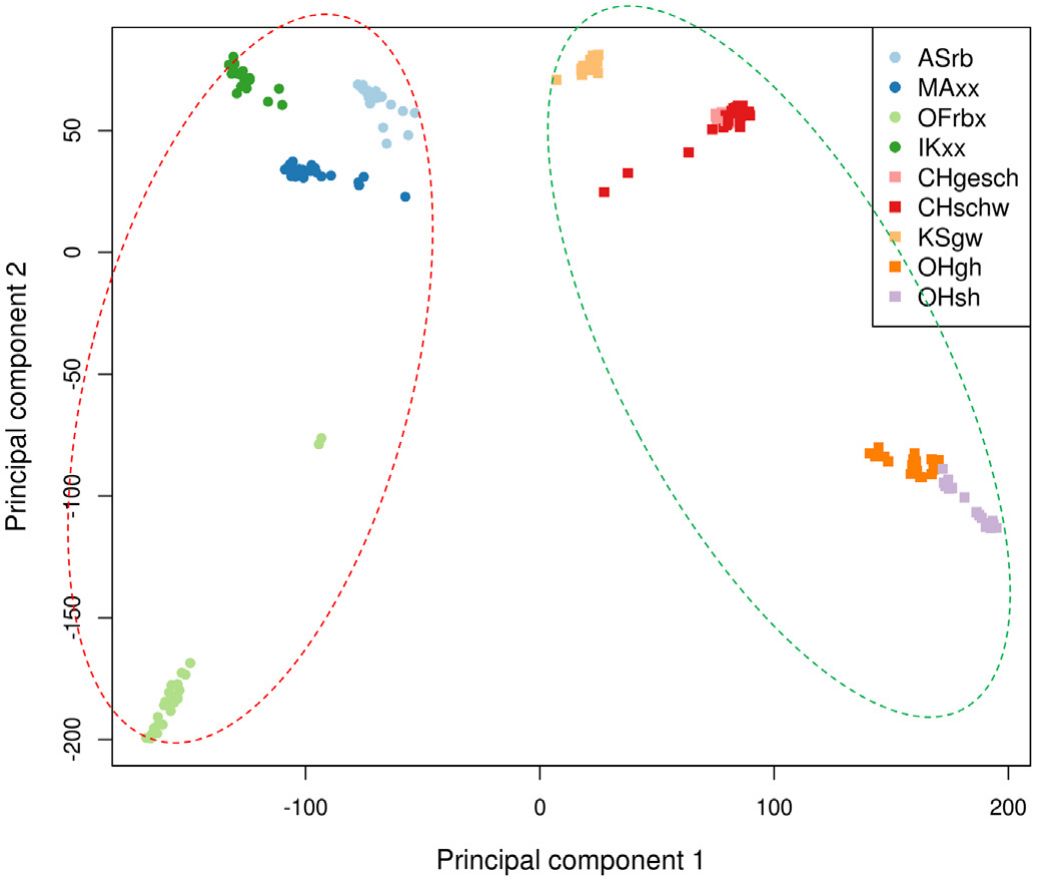
Principal component (PC) analysis showing PC1 versus PC2. Clear separation was observed between Asian Game (in red dashed circle) and Asian Bantam chickens (in green dashed circle) on PC1.

PC1 explained 13% of the genetic variation and separated the chickens into 2 clusters: Asian Game and Asian Bantam types (Figure 3). This is consistent with the results of the hierarchical clustering analysis. PC1 differentiates between the 2 chicken types clearly, and further Spearman's correlation analysis showed that the coordinates of the individuals on PC1 were significantly (|r| ≥ 0.77, *P*-value ≤ 2.24e-40) correlated with all the measured morphological traits (Table S2). This indicates that PC1 associates strongly with the difference in body size between the examined Game and Bantam type chickens. In total, 73 SNPs with significant contribution to PC1 were found after Bonferroni correction, which are located in 11 candidate regions (Table 3). The region on GGA4 from 20,113,606 to 20,379,905 bp overlapped with the GGA4 regions for keel length and body weight that were identified in the GWAS. Furthermore, seven out of 11 regions on GGA1, 2, 7, 14, and Z overlapped with previously reported QTLs (Table 3).

**Table 3:** Genomic regions identified by principal component analysis.

Top SNP^1^	Region^2^	Minor allele	Major allele	MAF^3^	Raw *P*-value	P_Bonf_^4^	Overlap with QTLs published in QTLdb^5^
rs317999764	1: 64288915 - 65870094	T	C	0.41	1.00E-07	0.017	Body weight (62,153, 62,163), Feather pecking (137,307, 137,308), Egg weight (63,779, 63,929), Egg number (64,486), Egg shell strength (57,879, 57,888), Egg shell thickness (57,889, 57,896), Egg shell weight (57,898, 57,910), Yolk weight (62,007), Ovarian follicle weight (62,014, 62,016), Ovary weight (62,017, 62,023), Intramuscular fat (62,096, 62,097), Egg shell color (37,926)
rs317769208	1: 72486629 – 72576268	G	T	0.49	7.94E-09	0.001	-
rs315645218	2: 45148419 – 46360352	C	T	0.49	1.58E-08	0.003	Body weight (95,404, 95,412), Egg shell strength (24968), Feather-crested head (127,112), Wattles weight (127,117)
rs317007003	4: 20113606 - 20379905	T	G	0.43	2.51E-08	0.004	-
rs317717388	7: 6976467 - 8981201	T	C	0.5	7.94E-09	0.001	Feed conversion ratio (139,741), Abdominal fat percentage (14,504), Comb weight (127,115), Feather pecking (137367)
rs313910027	14: 11892340 - 11901609	C	T	0.49	2.00E-09	0.0003	Wattles length (127,121)
rs315783766	20: 438802 - 441494	T	G	0.44	1.58E-07	0.027	-
rs317391544	Z: 11370833 - 11685040	A	T	0.49	3.16E-10	0.00005	Body weight (136,476, 136,865), Growth (136,610), Shank length (136,895), Egg shell strength (24,936)
rs313366229	Z: 18044764 - 19994683	T	A	0.41	1.26E-07	0.022	Body weight (136,324, 136,499), Growth (136520, 136,635), Shank length (136,905, 136,993), Intramuscular fat (24,484), Thymus weight (21,824)
rs317812756	Z: 50692623 - 51927985	T	G	0.45	1.00E-08	0.002	Feed intake (64,577), Earlobe color (108,451, 108,729)
rs16118569	Z: 63378841 - 63380441	A	G	0.48	1.58E-07	0.027	-

1 The SNP with the most significant contribution to principal component 1 (PC1).

2 The defined region associated with the difference in body size between Asian Game and Bantam chickens. SNPs contributing significantly to PC1 were merged into a region if they are 2 Mb of each other. The Regions were shown as “chromosome: region start position (bp) – region end position (bp)”. The locations are based on Galgal5 (ensembl 91).

3 MAF: minor all frequency of the top SNP.

4 The adjusted *p*-value using Bonferroni correction for multiple testing.

5 Previously identified QTLs with records in the Animal QTL Database (https://www.animalgenome.org/cgi-bin/QTLdb/index). If there are more than two QTLs mapped for one trait, only two were listed with the QTL ID.

### Investigation of Positional Candidate Genes

Since the region on GGA4 from 17,284,783 to 21,270,248 bp (Galgal5, Ensembl 91) was not only associated with keel length and body weight in the GWAS but also contained SNPs that significantly contributed to PC1 separating Asian Game and Asian Bantam type chickens, we focused on genes in this region. The functional annotation of the 60 genes (38 protein coding genes, 16 lincRNA, 5 miRNA, and 1 snRNA) located in this region yielded the 2 genes encoding myotubularin 1 (*MTM1*, GGA4: 17,820,524 - 17,851,478 bp) and secreted frizzled-related protein 2 (*SFRP2*, GGA4: 20,227,180 - 20,230,889 bp) having an effect on muscle and bone development, respectively (Table S3). Thus, *MTM1* and *SFRP2* were considered as candidate genes for growth differences between the examined chicken breeds that might influence muscle and bone development, respectively. *MTM1* is a positive regulator of skeletal muscle tissue growth (GO:0048633). *SFRP2* affects bone morphogenesis (GO:0060349) and is a positive regulator of osteoblast differentiation (GO:0045669).

## DISCUSSION

In this study, 9 chicken breeds belonging to 2 body size types; Asian Game and Asian Bantam, were used to investigate genomic regions associated with body size related-traits. Sixteen genomic regions were identified using GWAS to be significantly associated with wing length, shank thickness, keel length, and body weight. PCA identified 11 regions possibly related to the difference in body size between the 2 Asian chicken types. Combining the results of the 2 approaches, an overlapping region was found on GGA4 between 17,284,783 and 21,270,248 bp (Galgal5, Ensembl 91).

The overlapping region on GGA4 was associated with keel length and body weight. Since keel length is a bone trait, it suggests that this genomic region might contain genes affecting bone development. Additionally, this region is close (680 Kb downstream) to a QTL for tibia dry matter weight that was found in an F_2_ intercross population of 2 meat-type chicken lines divergently selected for high and low digestive efficiency (Mignon-Grasteau et al., 2016). The genetic linkage between a gene affecting growth and a gene affecting digestive efficiency is particulatly interesting since selection for growth efficiency would be superior to just body weight and growth. Since data for feed efficiency in our breeds were not available, it remains a task to examine the phases of linkage disequilibrium for favorable alleles in each breed. Within the identified GGA4 region, the gene *SFRP2* is known to play a role in bone development (Satoh et al., 2008). As a member of the secreted frizzled-related protein family, *SFRP2* acts as a regulator of Wnt signaling by interacting directly with Wnt ligands (Satoh et al., 2008). In human, Wnt signaling has an essential role in the differentiation and proliferation of osteoblast (Baron et al., 2013). Therefore, further investigation of the *SFRP2* gene could elucidate the underlying mechanisms of the physiological differences of bone development between the different chicken breeds. Another candidate gene in the GGA4 region, *MTM1* could contribute to the difference in body weight in our populations by influencing muscle growth and cell differentiation. In human, mutations in *MTM1* are responsible for the X-linked myotubular myopathy (Laporte et al., 1997; Schara et al., 2003; Bertazzi et al., 2015).

Both genes (*SFRP2* and *MTM1*) are expressed in the chicken brain and in the skeletal muscle (experiment E-MTAB-3724 in the “Expression Atlas” database). Bone expression data for chickens are not publicly available, nevertheless, in mice; *SFRP2* is highly expressed in the osteoblast (probeset of 1448201_at in “BioGPS” database). In chicken, *SFRP2* is involved in embryogenesis; development of the neural system (brain tissue) and muscles (myogenesis) (Lin et al., 2007). However, other genes in the identified GGA4 region might also have effects on growth, which have not been known yet, and therefore, cannot be excluded.

Our results suggest that at least 2 independent growth QTLs are present on GGA4; one between 17.3 Mb and 21.3 Mb, which has been detected in this study (Galgal5, Ensembl 91) and another one that previously has been identified in a region between 74.5 and 78.0 Mb (Galgal5, Ensembl 91, Lyu et al., 2017) in an intercross population between New Hampshire and White Leghorn chickens (Nassar et al., 2015).

Besides the region on GGA4, additional 15 and 10 regions were identified in the GWAS and PCA, respectively. The overlap between these regions and published QTLs (Hu et al., 2012) provided additional evidence that these regions are associated with chicken growth. For GWAS, the region on GGA6 between 10,926,588 and 10,933,626 bp which affected keel length in our study was reported to be associated with feed conversion ratio in broilers (QTL_ID in QTLdb: 139402), tibia bone mineral content, and tibia volume in an F_2_ cross between 2 lines of meat-type chickens selected for high or low digestive efficiency (QTL_ID in QTLdb: 135879 and 135882). The region on GGA10 from 4,534,620 to 7,754,637 bp for shank thickness was associated with body weight and blood glucose level in a Broiler × Fayoumi advanced intercross lines (QTL_ID in QTLdb: 95422 and 71191). The region on GGA27 from 3,375,301 to 3,375,436 bp for body weight was associated with femur bone mineral content and femur weight in an F_2_ cross between a Chinese indigenous breed and White Leghorn (QTL_ID in QTLdb: 130479 and 130480). With respect to PCA, which analyses the genetic diversity, seven regions on GGA1, 2, 7, 14, and Z overlapped with previously reported QTLs for body weight, feed conversion ratio, feed intake, egg laying performance, feather pecking, and earlobe color. PC1 likely also captures genetic variation that is associated with other traits than growth differences between Asian Game and Bantam type chickens. This is supported by regions that were detected in PCA and that are overlapping with QTLs for traits such as egg laying performance, feather pecking, comb shape, and wattles weight. Therefore, the regions identified by PC1 might contribute to the identification of genetic factors underlying other morphology traits besides the body weight trait investigated here.

In the GWAS, breed was considered as a fixed effect to correct for the population structure. However, the genomic inflation factor lambda (1.12 ≤ λ ≤ 1.35) was indicative for either an additional hidden subpopulation structure within breeds and/or the occurrence of several SNPs significantly associated with the trait under inspection. Considering the breed as a fixed effect in the model, some loci might be missed, namely SNPs that are correlated with the population structure itself, for example, regions harboring genetic variants that occur in a single population/type only or regions harboring variants with different genetic effects in different breeds. Therefore, an association analysis within each breed or in crosses between those breeds could help to identify additional genomic loci associated with body size variation within a specific breed. However, this breed-specific analysis would always require a larger sample size, as well as segregation of the specific locus either in the particular breed or, if the locus is fixed in this breed, in a cross-bred population with another breed to detect significant associations.

In conclusion, our study using genome-wide SNP data showed that significant genetic differences exist between Asian Game type (Aseel red mottled, Malay black red, Orloff red spangled, and Indian Game dark) and Asian Bantam type chickens (Japanese Bantam black tailed buff, Japanese Bantam black mottled, Ko Shamo black-red, Ohiki red duckwing, and Ohiki silver duckwing). GWAS identified 16 genomic regions, associated with chicken wing length (2), shank thickness (6), keel length (5), and body weight (3). Only 3 of the 16 regions overlapped with previously identified growth QTLs. This shows the value of investigating highly diverse breeds for identifying novel genomic regions involved in chicken size, including growth bone and muscle development. Based on PCA we identified SNPs in 11 genomic regions that significantly contribute to the difference in body size between Asian Game and Asian Bantam type chickens. Nevertheless, our study cannot exclude that some of these regions affect other traits which are consistently different between the 2 chicken types. The region on GGA4 between 17.3 and 21.3 Mb (Galgal5, Ensembl 91) was identified in GWAS and PCA. This shows that the PCA method is able to identify similar regions as GWAS, but is also able to detect regions which do not show a direct association with any of the morphological traits measured. Further investigation of positional candidate genes within the GWAS and PCA overlapping GGA4 region suggested *MTM1* and *SFRP2* as interesting candidate genes for the differences between Asian Game and Asian Bantam chickens with regards to muscle and bone development. However, effects of other genes in the GGA4 region on body size cannot be excluded.

## Appendix Supplementary materials

Image, application 1

Image, application 2

Image, application 3

Image, application 4

## Footnotes

Supplementary material associated with this article can be found in the online version at doi:10.1016/j.psj.2022.102303.
