# Supplementary material for: High-density genotyping reveals candidate genomic regions for chicken body size in breeds of Asian origin
Source: Poult Sci. 2022 Oct 29;102(1):102303. doi: 10.1016/j.psj.2022.102303 (PMC9706647; doi:10.1016/j.psj.2022.102303)

**Figure S1.** Representative chicken for each breed.


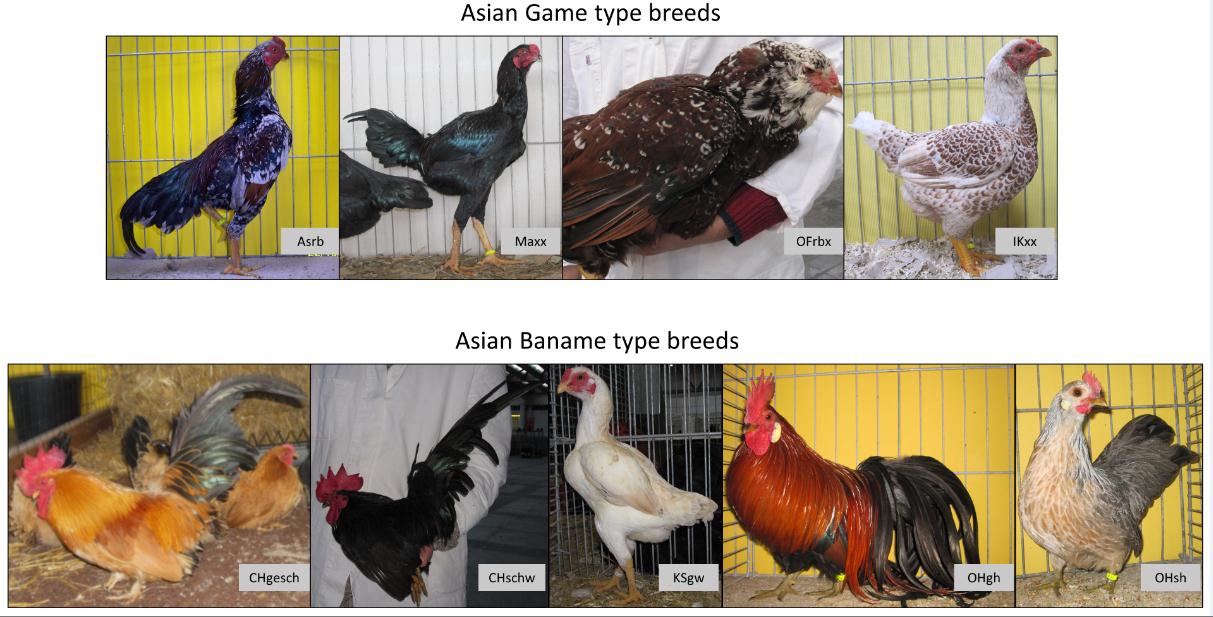


**Figure S2.** Phenotypes of Asian Game type and Asian Bantam type chickens (mean + standard deviation). **(a)** Males; **(b)** Females. Groups were tested for significant differences using the two-tailed Student's t-test. *** *p* < 0.0001.


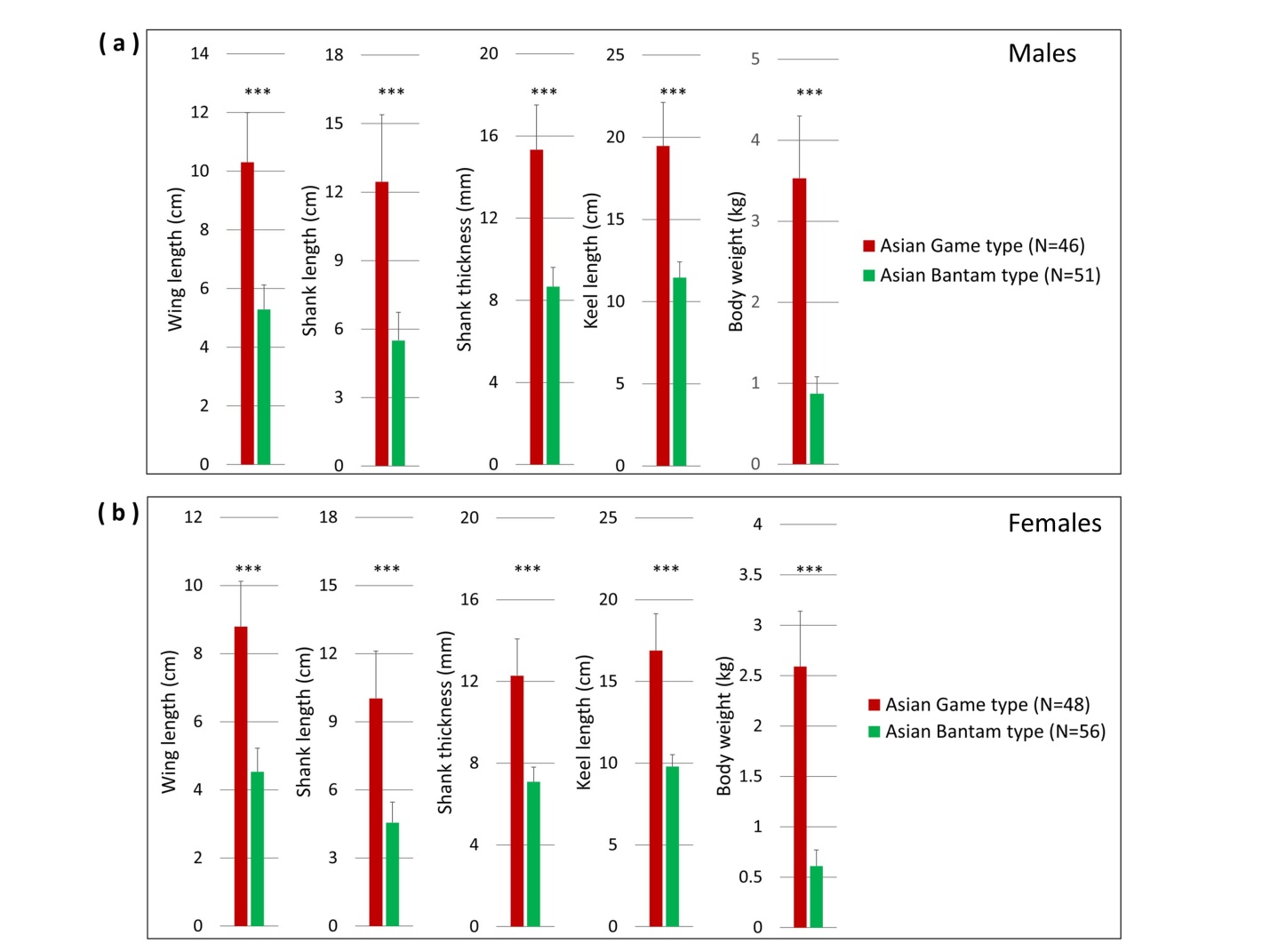

Supplement: Supplementary file 1 [file mmc1.docx]
