# Supplementary material for: High-density genotyping reveals candidate genomic regions for chicken body size in breeds of Asian origin
Source: Poult Sci. 2022 Oct 29;102(1):102303. doi: 10.1016/j.psj.2022.102303 (PMC9706647; doi:10.1016/j.psj.2022.102303)
Supplement: Supplementary file 2 [file mmc2.docx]

**Table S1** Phenotypic data of different chicken populations

| 1. Phenotypic data for males | | | | | | | | | |  |  |
| --- | --- | --- | --- | --- | --- | --- | --- | --- | --- | --- | --- |
| Trait^1^ | Breed^2^ | | | | | | | | | Type^3^ | |
|  | Asrb  (N = 9) | MAxx  (N = 15) | OFrbx  (N = 12) | IKxx  (N = 10) | Chgesch  (N =10) | CHschw  (N=13) | KSgw  (N=10) | OHgh  (N=8) | OHsh  (N=10) | Asian Game  (N=46) | Asian Bantam  (N=51) |
| Wing length (cm) | 8.03 ± 0.40 | 12.02 ± 0.46 | 11.11 ± 0.31 | 8.79 ± 0.41 | 4.30 ± 0.25 | 4.83 ± 0.48 | 6.41 ± 0.11 | 5.62 ± 0.36 | 5.5 ± 0.65 | 10.30 ± 1.69 | 5.29 ± 0.83 |
| Shank length (cm) | 9.05 ± 0.47 | 15.84 ± 0.84 | 13.20 ± 0.58 | 9.52 ± 0.71 | 4.30 ± 0.26 | 4.67 ± 0.57 | 7.42 ± 0.28 | 5.74 ± 0.40 | 5.69 ± 0.96 | 12.45 ± 2.94 | 5.50 ± 1.23 |
| Shank thickness (mm) | 13.12 ± 1.27 | 15.85 ± 1.34 | 13.85 ± 0.57 | 18.33 ± 0.93 | 8.75 ± 0.45 | 8.71 ± 0.57 | 9.99 ± 0.77 | 7.73 ± 0.46 | 7.90 ± 0.32 | 15.33 ± 2.18 | 8.66 ± 0.94 |
| Keel length (cm) | 15.42 ± 0.75 | 21.72 ± 1.17 | 20.92 ± 0.85 | 18.00 ± 1.22 | 10.54 ± 0.57 | 10.87 ± 0.66 | 12.01 ± 0.42 | 12.11 ± 1.03 | 12.08 ± 0.77 | 19.47 ± 2.65 | 11.46 ± 0.96 |
| Body weight (kg) | 2.23 ± 0.38 | 4.01 ± 0.41 | 3.57 ± 0.28 | 3.95 ± 0.47 | 0.80 ± 0.15 | 0.78 ± 0.14 | 1.12 ± 0.16 | 0.84 ± 0.29 | 0.83 ± 0.19 | 3.53 ± 0.77 | 0.87 ± 0.21 |
| 1. Phenotypic data for females | | | | | | | | | |  |  |
| Trait | Asrb  (N = 10) | MAxx  (N = 15) | OFrbx  (N = 13) | IKxx  (N = 10) | Chgesch  (N =12) | CHschw  (N=16) | KSgw  (N=10) | OHgh  (N=10) | OHsh  (N=8) | Asian Game  (N=48) | Asian Bantam  (N=56) |
| Wing length (cm) | 7.29 ± 0.26 | 10.16 ± 0.75 | 9.39 ± 0.22 | 7.47 ± 0.45 | 3.70 ± 0.20 | 4.36 ± 0.53 | 5.53 ± 0.19 | 4.76 ± 0.33 | 4.60 ± 0.32 | 8.79 ± 1.33 | 4.53 ± 0.69 |
| Shank length (cm) | 8.11 ± 0.16 | 12.44 ± 0.90 | 10.62 ± 0.37 | 7.58 ± 0.49 | 3.66 ± 0.23 | 4.17 ± 0.65 | 6.06 ± 0.25 | 4.7 ± 0.32 | 4.61 ± 0.46 | 10.03 ± 2.08 | 4.56 ± 0.90 |
| Shank thickness (mm) | 10.66 ± 0.50 | 12.86 ± 1.10 | 10.86 ± 0.35 | 14.87 ± 0.88 | 7.28 ± 0.27 | 7.01 ± 0.43 | 8.19 ± 0.57 | 6.45 ± 0.26 | 6.42 ± 0.45 | 12.28 ± 1.80 | 7.09 ± 0.72 |
| Keel length (cm) | 13.89 ± 0.67 | 18.95 ± 1.25 | 18.09 ± 0.59 | 15.25 ± 0.99 | 9.22 ± 0.46 | 9.68 ± 0.56 | 10.62 ± 0.53 | 9.81 ± 0.68 | 9.79 ± 0.81 | 16.89 ± 2.25 | 9.79 ± 0.73 |
| Body weight (kg) | 1.80 ± 0.24 | 3.06 ± 0.46 | 2.62 ± 0.22 | 2.64 ± 0.21 | 0.50 ± 0.07 | 0.61 ± 0.18 | 0.79 ± 0.10 | 0.65 ± 0.12 | 0.54 ± 0.14 | 2.59 ± 0.55 | 0.61 ± 0.16 |

**^1^**Wing length: measure from the elbow to the carpus where the little bone moves slightly; Shank length: measure from the fold in the foot pad to the hock joint; Shank thickness: measure the shank thickness at the point right above the spur; Keel length: measure from the tip of the chondral across the keel-bone towards the sternum where the bones of the clavicle form a triangle (Y-shaped wishbone).

^2^Asian Game type breeds: Aseel red mottled (ASrb), Malay black red (MAxx), Orloff red spangled (OFrbx) and Indian Game dark (IKxx); Asian Bantam type breeds: Japanese Bantam black tailed buff (CHgesch), Japanese Bantam black mottled (CHschw), Ko Shamo black-red (KSgw), Ohiki red duckwing (OHgh) and Ohiki silver duckwing (OHsh). N corresponds to the sample size of each breed. Data were shown as “Mean ± standard deviation (SD)”.

**^3^**The mean and SD for Asian Game type breeds and Asian Bantam type breeds, respectively.
