# Supplementary material for: High-density genotyping reveals candidate genomic regions for chicken body size in breeds of Asian origin
Source: Poult Sci. 2022 Oct 29;102(1):102303. doi: 10.1016/j.psj.2022.102303 (PMC9706647; doi:10.1016/j.psj.2022.102303)
Supplement: Supplementary file 3 [file mmc3.docx]

**Table S2** Spearman’s correlation analysis between phenotypes and coordinates of all individuals on the first principal component.

| Trait | r | *p*-value |
| --- | --- | --- |
| Wing length | -0.78 | 8.10e-43 |
| Shank length | -0.77 | 2.24e-40 |
| Shank thickness | -0.87 | 1.01e-63 |
| Keel length | -0.80 | 3.96e-45 |
| Body weight | -0.81 | 2.05e-48 |
